# Hormonal regulation of circuit function: sex, systems and depression

**DOI:** 10.1186/s13293-019-0226-x

**Published:** 2019-02-28

**Authors:** Rachel-Karson Thériault, Melissa L. Perreault

**Affiliations:** 1 Department of Molecular and Cellular Biology, University of Guelph (ON), 50 Stone Rd. E, Guelph, Ontario N1G 2W1 Canada; 2 Collaborative Neuroscience Program, University of Guelph (ON), Guelph, Canada

**Keywords:** Network function, Neuronal oscillations, Depression, Sex, Sex hormones, Female cycling

## Abstract

Major depressive disorder (MDD) is a debilitating chronic illness that is two times more prevalent in women than in men. The mechanisms associated with the increased female susceptibility to depression remain poorly characterized. Aberrant neuronal oscillatory activity within the putative depression network is an emerging mechanism underlying MDD. However, innate sex differences in network activity and its contribution to depression vulnerability have not been well described. In this review, current evidence of sex differences in neuronal oscillatory activity, including the influence of sex hormones and female cycling, will first be described followed by evidence of disrupted neuronal circuit function in MDD and the effects of antidepressant treatment. Lastly, current knowledge of sex differences in MDD-associated aberrant circuit function and oscillatory activity will be highlighted, with an emphasis on the role of sex steroids and female cycling. Collectively, it is clear that there are significant gaps in the literature regarding innate and pathologically associated sex differences in network activity and that the elucidation of these differences is invaluable to our understanding of sex-specific vulnerabilities and therapies for MDD.

## Background

Major depressive disorder (MDD), a highly prevalent chronic illness, is the leading cause of disability worldwide, affecting about 10% of the world population [1]. Depression currently has the third highest burden of disease worldwide and is predicted to be the leading cause by 2030 [2]. In women, MDD is already the leading cause of global disease burden [2] with a prevalence almost two times higher than in men [3]. The mechanisms associated with the increased female vulnerability to depression most likely involve sex hormones. Yet, less is known regarding the specific physiological processes that are impacted through hormonal regulation that confer this susceptibility.

Evidence of sex-dependent neurocircuitry function and altered network activity in MDD suggest that circuit function regulation may be an important contributing factor in the female vulnerability to this disorder. A key principle in neuroscience research is that brain circuit changes, derived from changes in activity of neuron populations, have physiological patterns that are highly conserved across species, and are coupled to specific behavioral states [4]. As such, the dysregulation of circuit function has been widely shown to play an integral role in various neuropsychiatric disorders such as schizophrenia, where a pivotal role for neuronal network dysfunction in mediating the negative and cognitive symptoms of the disorder has been documented [4]. In MDD, deficits in network function, such as frontal and parietal lobe asymmetries [5], have also been suggested to contribute to depression symptoms. Yet, there has been little focus on the contribution of network abnormalities to differences in depression susceptibility between men and women. There is therefore a substantial need for additional research focused on sex-specific mechanisms in depression pathology, especially in the area of preclinical research, where male animals are predominantly used in order to limit data variability that may arise as a result of hormone fluctuations. Closing the knowledge gap of innate and pathological sex differences will bring us closer to developing more effective personalized treatment strategies, as well as minimizing disease burden on an individual and global scale.

This review will discuss baseline sex differences in neuronal circuit function and the influence of sex hormones on oscillatory activity. Following, evidence of disrupted network function in MDD will be highlighted as well as the effect of antidepressant treatment. Lastly, current knowledge of sex differences in MDD-associated aberrant circuit function and oscillatory activity will be discussed, with an emphasis on the role of sex steroids and female cycling.

## Sex differences in circuit function activity and regulation

There is widespread evidence that neuronal oscillations are a fundamental mechanism in the coordination of neuronal responses during normal brain activity and the maintenance of healthy circuit function [4]. Synchronous activity of neuronal ensembles [6] generates macroscopic oscillations, which give rise to brain electrophysiological rhythms that are critical for normal neuronal communication within and between brain regions [4, 6]. Ensembles of neurons can coordinate their rhythmic activity to generate synchronous activity, termed coherence, which can occur within a range of oscillatory frequencies: the low frequency bands (delta (0.5–4 Hz), theta (> 4–8 Hz), alpha (> 8–12 Hz)) are slow waves and are critical in long-distance or between region communication, whereas the high frequency bands (beta (> 12–30 Hz), gamma (> 30 Hz)) are fast waves and are involved in short-distance or within region communication [7, 8]. Coherence between neuronal ensembles is thought to reflect effective communication as their input and output communication windows are open at the same time; a hypothesis first postulated by Fries known as communication-through-coherence [7]. For a more comprehensive review on neuronal oscillations and specific frequency band functions, see [4, 8], respectively.

The measurement and correlation of neuronal oscillatory activity are most often achieved through electroencephalography (EEG), magnetoencephalography (MEG), or multielectrode arrays, and can elucidate the overall degree of network synchrony. The majority of current knowledge and evidence discussed in this review is derived from studies using quantitative EEG, a useful tool in elucidating regional and global network changes. Although, it is notable that this method cannot be used to evaluate activity and function in specific brain structures, particularly those underlying cortical structures.

EEG studies in human subjects have been of critical importance in highlighting sex differences in both the resting state and task-induced network activity of adults [9–13]. When examining overall brain electrical activity in brain structures, women exhibited higher EEG power at rest and during sleep [12, 13], less power asymmetry between hemispheres while performing spatial and verbal tasks, and a lower degree of local cortical specialization of functions [9, 14], in comparison to men. Larger interhemispheric coherence has also been noted in women at rest and during cognitive activity and is indicative of greater similarity in the EEG patterns in both hemispheres [9, 11, 12]. Across all frequency bands, women exhibited significantly greater interparietal coherence, reflective of lower hemispheric specialization in verbal and spatial processing in these regions for women, while the opposite pattern is observed in men [11]. Evidence also suggests that there is a significant correlation between interhemispheric coherence and Differential Aptitude Test score, a measure of verbal, spatial, and abstract reasoning [10]. Notably, men and women had opposite signs of correlation; that is, higher scores in men were correlated with lower interhemispheric coherence, while higher scores in women were associated with higher interhemispheric coherence, suggesting the presence of sex-dependent differences in cerebral functional organization [10]. Within specific frequency bands, women exhibited higher alpha relative power (the percent power in relation to total power), possibly reflective of greater information processing, whereas men had higher beta relative power, a finding potentially related to a greater reactivity to the environment and better coping abilities [11]. Additionally, only men distinctly showed higher beta relative power in the left over the right parietal region; however, the direct behavioral implications of this are unknown [11].

Animal models have also been invaluable in further elucidating sex differences in network activity. In line with clinical studies in humans, studies measuring EEG activity in rats found that males exhibited greater cortical functional asymmetry and higher right over left parietal coupling than females, indicative of increased cooperativity between hemispheres [15]. Interhemispheric correlation in the theta band and global theta relative power were also significantly greater in male rats at rest and during exploratory behavior, a finding that represents enhanced functional coupling, most likely in the hippocampus (HIP) where theta oscillations are generated [15, 16]. Male rats also showed greater interhemispheric delta and alpha coherences, while female rats had greater global delta and beta relative power; however, the functional relevance of these sex differences is not known [15, 16]. Notably, no studies to date have used a non-human primate model to validate these findings and present a significant gap in the current literature. These sex differences in network activity within the various species likely reflect, at least in part, differences in sex steroid as these hormones have been shown to directly regulate circuit function.

### The modulation of network activity by sex steroids

Attempts to delineate the mechanistic role of sex hormones in regulating the activity of neuronal circuitry have most commonly come from animal studies where these hormones can be directly manipulated. For instance, following puberty, gonadectomized (GNX) rats no longer showed right over left parietal activation or the aforementioned sex differences in interhemispheric coherence, and theta and delta relative power [17, 18]. GNX male rats exhibited higher overall absolute power (raw power value recorded) than females, a sex difference that is not present in healthy rats [18]. However, when males were postnatally GNX, they exhibited more feminized EEG features, such as enhanced relative beta power, indicating that testicular steroids are essential for the masculinization of brain activity during the neonatal period [17]. Similar findings have been reported by studies that used prenatal and postnatal testosterone propionate (TP) treatment as an alternative to GNX, as it disrupts normal sexual differentiation and induces virilization [19–21]. Prenatal TP treatment abolished sex differences and increased the absolute power of all frequency bands, with the exception of the beta band, in both sexes [16, 17]. TP administration prenatally also masculinized the network activity of female rats, as evidenced by greater theta absolute power, reduced beta relative power, and enhanced interparietal correlation [16, 17]. These results indicate that sex differences in oscillatory activity are established during this critical period of sexual differentiation, likely through the organizational effects of sex steroids. In contrast, postnatal TP administration in female rats increased beta power and decreased interparietal correlation [17], demonstrating that sex steroids differentially influence network activity pre- and postnatally in females. Postnatal virilization not only masculinized female rat EEG patterns, but also masculinized their behaviors, including social play-fighting and sexual behavior [19–21]. Collectively, these findings support the necessity of both the organizational and activational effects of sex steroids for the neuronal mechanisms underlying the sex differences in EEG patterns, which likely underlie the expression of behaviors in a sex-dependent manner.

This is further corroborated by the fact that hormonal treatment post-adult GNX (estradiol or progesterone in female rats, TP or 5α-dihydrotestosterone (5α-DHT) in male rats) re-established the lack of sex differences in absolute power, as well as the interparietal asymmetry in both sexes [18]. Although, it is important to note that hormonal treatment did not re-establish the sex differences in interhemispheric coherence or the relative power of delta and theta bands [18]. Therefore, it appears as though sex hormones may modulate EEG patterns only within specific brain sites. Moreover, progesterone and estradiol treatments were not given simultaneously, which may have contributed to the lack of re-established activity, as these hormones likely have synergistic effects in intact females [18]. According to Fernandez-Guasti et al., the effects of progesterone are sexually dimorphic, but do not depend on the sexual differentiation process that occurs postnatally [22]. Thus, progesterone increased both the absolute power of the alpha and beta band and the interparietal correlation of the alpha band in both intact and postnatally GNX male rats, whereas in female rats, intact or postnatally virilized, progesterone reduced the interparietal correlation in both the theta and alpha frequencies [22]. These alterations may be associated to sex differences in progesterone metabolism and/or receptor expression or the reported anxiolytic effects of progesterone, as males have a higher sensitivity to respond to anxiolytic drugs [23–25].

Although these studies provide evidence that sex steroids are critical for the development and maintenance of sex differences in inherent network activity and subsequent behaviors, there remains a significant gap in preclinical research regarding sex differences and the sex hormone-mediated mechanisms regulating system functions in the brain. Major findings of the sex differences in circuit function and the influence of sex hormones are summarized in Table 1.

**Table 1:** Sex differences in circuit function and the influence of sex hormones

Study	Species	Major findings
Beaumont et al. [9]	Adult humans	Women had less power asymmetry between hemispheres during spatial and verbal tasks, a lower degree of cortical specialization of functions, and larger interhemispheric coherence, compared to men.
Corsi-Cabrera et al. [10]	Adult humans	Men had a negative correlation between interhemispheric coherence and an aptitude test score for verbal, spatial, and abstract reasoning, while women had a positive correlation.
Corsi-Cabrera et al. [11]	Adult humans	Women had greater interparietal coherence across all frequency bands and higher global alpha relative power, while men had higher global beta relative power and greater beta relative power in the left over the right parietal region.
Juarez and Corsi-Cabrera [15]	Adult Wistar rats	Interhemispheric theta coherence and global theta relative power were greater in males. Males showed greater interhemispheric delta and alpha coherences, while females had greater global delta and beta relative power.
Juarez et al. [16]	Adult Wistar rats	Males had greater cortical asymmetry and higher right over left parietal coupling than females. Interhemispheric coherence in delta and theta bands was greater in males. Females had a greater global delta and beta relative power and a lower global theta relative power, compared to males.
Corsi-Cabrera et al. [17]	Adult Wistar rats pre- or postnatally virilized	Postnatally GNX males had increased global beta relative power. Prenatal TP treatment abolished sex differences and increased the absolute power of all frequency bands, except beta, in both sexes, as well as enhanced theta absolute power, reduced beta relative power, and increased interparietal coherence in females. Postnatal TP treatment increased beta power and decreased interparietal coherence in females.
Del Rio-Portilla et al. [18]	Adult GNX Wistar rats	GNX abolished baseline sex differences and induced higher overall absolute power in males than females. Hormonal treatment re-established the interparietal asymmetry in both sexes and the lack of sex differences in absolute power. Hormonal treatment did not re-establish sex differences in interhemispheric coherence or relative delta and theta power.
Fernandez-Guasti et al. [22]	Adult intact or neonatally virilized Wistar rats	Progesterone increased absolute alpha and beta powers and the interparietal alpha correlation in both intact and virilized males. Progesterone reduced the interparietal theta and alpha correlations in both intact and virilized females.

*GNX* gonadectomized

### Influence of female cycling on oscillations

As the literature shows that the manipulation of sex hormones, including estradiol and progesterone, can influence neuronal circuit function, it follows that female cycling would also impact neuronal oscillatory activity. Studies measuring EEG activity in naturally cycling women have consistently shown a significant increase in alpha frequency waves during the luteal phase and a decrease in alpha frequency power during the follicular phase [26–31], a pattern that appears to be predominantly localized to the parietal region [27], a region involved in the processing of emotional stimuli [32]. Thus, as EEG alpha activity is inversely correlated to cortical reactivity [29], these results indicate that cortical circuit function is influenced by the fluctuations of estradiol and progesterone levels throughout the menstrual cycle, which may have a subsequent impact on mood. In line with this, a significant negative correlation between resting alpha frequency and endogenous estradiol levels in naturally cycling women has been reported [27, 28] and estradiol is strongly implicated in the regulation of mood and behaviors, with low estradiol levels correlating to lower mood [33]. Together, these findings might imply that periods of increased endogenous estradiol levels mediate an increase in cortical activity via alpha band oscillations, leading to the promotion of positive mood and emotions. With regard to progesterone, the correlation with the alpha frequency merits further investigation as a lack of significant correlation [29, 30] and a positive correlation [34] have both been reported.

The correlation between EEG activity in other frequency bands and cycling has been conflicting and requires further examination. With regard to theta, some reports have shown decreased [26, 30, 35] and increased [31] global theta power in naturally cycling women during the luteal phase. Similarly, there have been reports of no changes in beta oscillations across phases of the menstrual cycle [30], as well as reports of enhanced beta frequency power during menstruation [31] and decreased relative beta power during the luteal phase [31, 36, 37]. Increased delta power during the luteal phase has also been observed [31], particularly within the occipital region [38]; however, the implications of this change are not known. It was also shown that the connection between gamma-generating interneurons and pyramidal cells was stronger in the early follicular phase, compared to the mid-luteal phase, potentially marking fluctuations in theta-gamma coupling throughout the menstrual cycle [39].

Unfortunately, there is a significant lack of preclinical studies that have used animal models to investigate the influence of fluctuating sex hormones during female cycling on network oscillatory activity. However, there is support for reduced interhemispheric coherence during diestrus, as compared to proestrus and estrus [40]. Notably, it has recently been shown that gamma oscillations in the HIP are influenced by estrous cycling and estradiol levels. Within the CA1 region, gamma oscillations generated by parvalbumin (PV)-containing interneurons are reduced during diestrus and enhanced during estrus [41]. In contrast, these changes in gamma oscillations were not observed in non-cycling wild-type female rats or in cycling females with the delta subunits of GABA_A_ receptors knocked out in PV-containing interneurons [41], which further supports that interneuron-induced gamma oscillations are influenced by the estrous cycle. In line with this, Schroeder et al. investigated the link between estradiol and gamma oscillations in the dorsal HIP (dHIP) using ovariectomized (OVX) mice [42]. The authors found that OVX mice had deficits in spatial memory, in addition to a significant reduction in dHIP gamma oscillations during decision making in a spatial memory task [42]. However, treatment with either estradiol or raloxifene, a selective estrogen receptor modulator, rescued both the behavioral and electrophysiological deficits [42]. This was the first study to demonstrate that estradiol has direct effects on the generation of gamma oscillations in the dHIP, although this was not surprising as gamma-generating, PV-containing interneurons reportedly show co-expression with estrogen receptors, thereby allowing direct modulation of their function by estradiol [43].

Due to the drastic changes in sex hormone levels that occur during pregnancy and postpartum, the associated changes in network activity have also been investigated. Overall, there are no reported oscillatory differences between the third trimester of pregnancy and 6 months postpartum, which may suggest that the neuronal network adapts to maintain normal functioning in response to the significant changes in hormone levels [44]. This is supported by several in vitro studies that have demonstrated a compensatory reduction in delta subunit-containing GABA_A_ receptors in the HIP, striatum, and thalamus of pregnant mice, in response to sex hormone-induced changes in gamma power during pregnancy [45–47]. These anatomical changes revert to control levels within 48 h postpartum [45–47]. Thus, it appears that GABA_A_ receptor homeostatic plasticity is critical for normal network functioning during periods of significant changes in steroid levels [46]. Further, based on the link between perturbed gamma activity and psychiatric disorders, it is suggested that a dysfunction in this homeostatic mechanism during pregnancy or postpartum may induce aberrant network activity, thereby precipitating psychiatric conditions, such as postpartum depression [45]. Indeed, mice that did not properly regulate the expression of delta subunit-containing GABA_A_ receptors during pregnancy or postpartum exhibited depression-like behaviors during the postpartum period only [46, 47]. However, whether these findings translate to humans remains unknown.

## Aberrant network function in major depressive disorder

Aberrant network activity is an emerging common mechanism for many neuropsychiatric disorders, including MDD and anxiety. Specifically, the disturbance of neuronal networks in brain structures implicated in MDD appears to be associated with the emergence of depression symptoms; however, the actual mechanism and pathophysiological consequence of these disturbances are not well understood. The predominant MDD-associated changes in network activity are summarized in Table 2.

**Table 2:** Aberrant oscillatory activity in human patients with MDD

Frequency band	Reported changes in MDD patients
Delta	Reduced global power [95, 96] Increased global power [97, 98] Decreased phase synchronization in the centro-parieto-occipital regions [84]
Theta	Reduced power in the occipito-parietal region [88, 89] Aberrant synchronization in the frontoparietal network [89] Abnormal activity in corticolimbic structures [88, 89]
Alpha	Increased global power [48–50] Frontal asymmetry (L > R) [8, 55, 57–62] Parietotemporal asymmetry (R > L) [60, 65, 74–78] Decreased synchronization in frontoparietal network [84] Increased resting power in the NAc [86]
Beta	Dominant global power [83–85] Increased synchronization in frontoparietal network [84]
Gamma	Reduced power in the frontal and central regions [103]

### Alpha frequency

Quantitative EEG studies most commonly report disturbances in the alpha frequency oscillatory activity within many regions of the putative depression brain network. Patients with MDD exhibited a global increase in alpha power [48–50], thought to reflect cortical hypoactivity [51–53]. Consistent with this, the normalization of alpha oscillatory power in MDD has been found to be associated with a successful response to antidepressant treatment [54–56]. Moreover, an important marker of depressive symptoms appears to be frontal alpha asymmetry, with MDD patients showing increased left over right frontal alpha activity, indicative of reduced left over right frontal activity [8, 55, 57–62]. Importantly, this pattern has been found to distinguish both currently symptomatic patients and remitted depressed patients, from individuals with no history of depression [57, 59, 60, 63], and appears to positively correlate with depression severity scores [61, 64]. As such, asymmetry between the frontal cortices is hypothesized to be an endophenotype of depression and MDD susceptibility [58, 65].

Left frontal activity is thought to influence approach motivation in individuals, associated with positive affect, reward responsiveness, and the engagement with pleasant stimuli, while right frontal activity is related to withdrawal motivation, associated with negative affect and the avoidance of aversive stimuli, and for this reason, the marker of frontal asymmetry is often interpreted by the approach-withdrawal model [51, 63, 66, 67]. Several functional magnetic resonance imaging (fMRI) studies have substantiated this approach-avoidance lateralization and have specifically localized this system to the dorsolateral prefrontal cortex (PFC) [68, 69]. Moreover, when presented negative stimuli, fMRI showed a significantly greater activation of the right PFC of MDD subjects compared to healthy controls [68]. Thus, the frontal asymmetry pattern observed in MDD patients may reflect a reduction in left frontal-associated approach motivation and an increase in right frontal-associated withdrawal motivation. However, this interpretation gets complicated when co-morbid anxiety is taken into account. Some studies have reported that frontal asymmetry is more indicative of anxiety symptoms in the presence of depression, rather than depression itself [65, 70, 71]. In this regard, it is important to note that two types of anxiety have been identified: anxious apprehension (i.e., feelings of worry experienced in generalized anxiety disorders) and anxious arousal (i.e., panic disorders) [51, 72]. When examined separately, it appears that individuals that predominantly displayed anxious apprehension showed a left over right frontal asymmetry, whereas those that displayed anxious arousal showed a right over left frontal asymmetry, as is characterized in depression [72, 73]. This may indicate that anxious apprehension suppresses the right over left frontal activity in depression, whereas this finding is enhanced in depressed individuals with co-morbid anxious arousal.

Another potential endophenotype of MDD and depression vulnerability is increased alpha oscillations in the right over the left hemisphere in the parietotemporal sites, which reflects increased left over right parietal activity [60, 65, 74–78]. Evidence suggests that the right parietal cortex is associated with the processing of emotional stimuli [79], and therefore, reduced right parietal activity may cause impaired perception and processing of emotional information in depressed individuals [51]. Right parietal hypoactivity has also been suggested to contribute to deficits in social skills in MDD subjects [60]. Although not all EEG studies have found this characteristic parietal pattern [59, 73, 80], these discrepancies may be attributable to the presence of co-morbid anxiety. Evidence supports that reduced right parietal activity is seen in individuals with non-anxious depression, while anxious arousal is associated with increased right parietal activity, and therefore may mask any changes in parietal activity when co-morbid with depression [65, 70, 76]. No fMRI investigations have reported this parietal pattern in MDD subjects either; however, enhanced parietal activation in patients with MDD does appear to be indicative of remission and improved performance in working memory tasks [81, 82].

Additionally, there have been reports of increased parietotemporal alpha coherence [83, 84] and cortical synchrony within the brains of MDD subjects [85]. A reduction in alpha synchronization in the frontoparietal network of individuals with MDD has also been shown, which may directly contribute to impaired short-term memory retention [84]. Furthermore, resting alpha rhythms were found to be increased in the nucleus accumbens (NAc), the bed nucleus of stria terminalis, and the subgenual cingulate cortex of treatment-resistant MDD patients, relative to patients with obsessive-compulsive disorder (OCD) [86]. Despite the fact that these results should be confirmed against healthy control subjects, this was the first evidence implicating alpha dysfunction in limbic structures in the context of depression and may represent impaired emotional regulation [86].

### Theta frequency

Theta rhythms are functionally associated with attention, learning and memory, emotional processing, and cognition, functions that are generally impaired in MDD [87]. Subjects with depression are reported to have abnormal theta activity within corticolimbic networks and reduced theta oscillations in the occipital-parietal regions, which may reflect disrupted affect regulation and functional connectivity in the frontal-cingulate pathways [88, 89]. Additionally, theta dysfunction in depression was found to be linked to impaired memory retention and working memory, as depressed individuals showed aberrant theta synchronization in the frontoparietal network during a task of central executive functioning [84]. Thus, it is not surprising that the normalization of theta activity within the anterior cingulate cortex (ACC) is a reportedly reliable measure of treatment response [90, 91], as well as reduced theta cordance in the PFC [92, 93]. Furthermore, excessive baseline theta oscillations in the frontal-midline regions of the brain were predictive of a successful treatment response and improved verbal memory post-treatment [94].

### Delta frequency

With regard to oscillatory activity in the delta frequency band, there have been conflicting findings of reduced [95, 96] and elevated [97, 98] overall delta power. EEG studies that reported an increase in delta power postulate that this is a sign of a more subtle cognitive dysfunction, as individuals with dementia and delirium also exhibit an increase in delta power, but to a much larger degree [98]. Authors have also found that depressed individuals showed a less pronounced delta phase desynchronization in the centro-parieto-occipital regions and that this may cause inadequate cortical inhibition, thereby attenuating the synchrony of other frequency bands in the frontoparietal network [84].

### Beta frequency

Another prominent characteristic of subjects with MDD is the dominance of oscillations in the beta frequency band, particularly within the PFC and temporal regions [85], relative to all other frequency bands [83, 84]. Beta oscillatory activity also positively correlated with an individual’s probability of relapsing [99] and the number of depressive episodes [96]. In general, beta oscillations appear to be critical in attentional processes and the maintenance of current cognitive states [100, 101]. During a task of executive functioning, individuals with MDD exhibited an increase in beta synchronization in the frontoparietal network, indicative of enhanced attentional control and task engagement [84]. Thus, the beta frequency dominance observed in MDD is hypothesized to represent a compensatory mechanism for the symptoms of impaired memory retention and working memory [84]. Conversely, others have theorized that this beta band dominance may actually reflect impaired cognitive control [83].

### Gamma frequency

Gamma oscillatory activity in human patients with depression has not been extensively investigated [102]. Only recently has a reduction in gamma power within the frontal and central brain regions of subjects with depression been reported to correlate with the reduction in depression severity score post-treatment with the selective serotonin reuptake inhibitor (SSRI) paroxetine [103]. However, it is likely that gamma oscillations play a role in MDD given that certain subtypes of GABA-ergic interneurons, known to be pivotal to the generation of cortical and hippocampal oscillations [8, 104], have impaired function in patients with depression [105, 106]. Specifically, a significant reduction in the number of gamma-generating, PV-containing interneurons is observed in the postmortem PFC and HIP of subjects with MDD [107], as well as adult rats exposed to chronic stress [108, 109]. In line with this, selective suppression of PV-containing interneurons in the medial PFC of mice promoted helplessness in the learned helplessness model of depression [110], suggesting that stress-induced activation of these neurons contributes to resiliency [111]. Reduced expression of somatostatin (SST)-containing GABA-ergic interneurons has also been reported in the postmortem brains of human patients with MDD [112, 113] and mouse models of depression [114], with disinhibition of SST-containing GABA-ergic interneurons using Cre recombinase resulting in anxiolytic and antidepressant effects in mice, without impairing spatial learning and memory [115].

Not only does co-morbid anxiety complicate the findings of altered circuit function in MDD, as previously discussed, but it has also recently been shown that individuals with treatment-resistant depression exhibit circuitry changes distinct from treatment-responsive depression. Baskaran et al. compared the EEG profiles of treatment-responsive and treatment-resistant depression and found that patients with treatment-resistant depression had a left over right frontal asymmetry and greater delta power in the left hemisphere at baseline and 2-weeks post-treatment with escitalopram showed increased beta power in the right hemisphere, whereas treatment-responsive individuals displayed the opposite patterns [55]. Furthermore, an increase in frontal theta activity was predictive of treatment-resistant depression [91, 116], and when normalized through deep brain stimulation (DBS), produced 6 months of symptomatic relief [117]. Collectively, these findings suggest that treatment-resistant depression has a unique pathophysiological mechanism.

Additionally, it has recently come to light that the therapeutic effects of antidepressants are likely mediated, at least in part, via the modulation of neural oscillatory activity. Leuchter et al. investigated the effect of escitalopram treatment on prefrontal oscillatory activity in subjects with MDD using quantitative EEG. One week of escitalopram treatment enhanced the mean delta and theta power and decreased alpha power, an effect that predicted the probability of successful remission after 7 weeks of medication treatment [56]. Moreover, these changes were distinct from placebo groups, and thus, specific antidepressant-induced changes in oscillatory changes may act as a biomarker for clinical efficacy [56].

## Sex differences in the oscillatory activity of patients with MDD

The investigation of sex differences in network activity of patients with MDD may help us elucidate sex-specific mechanisms to more effectively understand and treat this affective disorder. Unfortunately, the number of studies in this field is limited, possibly resulting from the relatively inconsistent findings reported from these studies thus far. One example of this is the relationship between the frontal hemispheric asymmetry and depression. As previously mentioned, it is generally accepted that patients with MDD exhibit greater left than right frontal alpha band activity, indicative of left frontal hypoactivity and right frontal hyperactivity [57, 59, 60, 62, 63, 81, 118], and this is thought to reflect decreased responsiveness to reward and reduced motivation [62]. However, the robustness of this finding dissipates once the results are broken down by sex.

Overall, many clinical EEG studies have reported that the frontal alpha asymmetry is present in only women with MDD and not men with MDD or healthy controls [62–64, 67, 71, 119, 120]. Although these studies predominantly found greater right frontal activity over left [62, 64, 119, 120], a frontal asymmetry in the opposite direction for depressed women has also recently been reported [67]. Conversely, other studies have reported the presence of a frontal asymmetry in male MDD patients; however, these findings are conflicting. For example, Miller et al. found that depression in males was associated with more relative left frontal activity [119], whereas Jacobs and Snyder found the opposite pattern [121]. More recently, Jaworska et al. reported that both males and females with depression had left frontal hypoactivity [118], but that this effect was more pronounced in males, while a lack of difference in frontal asymmetry between depressed and non-depressed men has also been reported [67]. However, the authors noted that it is likely that methodological factors contributed to the differences in findings, such as the heterogeneity of the depressed samples [67] and the presence of co-morbid anxiety [65, 72, 120].

It also appears that only women show an association between frontal asymmetry and the severity of depression, in that women with more severe depressive symptoms exhibited greater left frontal hypoactivity, compared to women with mild symptoms, while women with moderate symptoms of depression had a left frontal activity in between the low and high group [62, 63, 71], thus further supporting that this oscillatory phenotype may be implicated in the expression of depressive symptoms [67]. In contrast, this pattern was not consistent for men with MDD [62]. In addition to depression severity, the normalization of the frontal asymmetry has been found to be indicative of successful response to fluoxetine, sertraline, and escitalopram, but not venlafaxine, only in depressed women [64, 116]. Conversely, women that did not respond to fluoxetine treatment exhibited a frontal asymmetry similar to MDD patients with co-morbid anxiety [64], further substantiating that this circuit characteristic may be specific to non-anxious female MDD patients.

The association between parietal asymmetry, depression, and sex has also received some investigatory attention, although the results have once again been inconsistent. Some studies have found that depressed women with and without co-morbid anxiety disorder showed right parietal hypoactivation [74, 76, 122], whereas others have observed no association between parietal activity and MDD [58, 123]. In contrast, Jaworska et al. found that women with MDD, and not men with MDD or healthy controls, showed increased right over left parietal activity, which may represent emotional hyperarousal in these patients [118, 124, 125], as evidence has linked right parietal hyperactivation to specific features of co-morbid anxiety, such as somatic symptoms [118, 126]. This hypothesis is supported by previous studies which have demonstrated that non-anxious and subclinically depressed patients exhibited right parietal hypoactivation [65, 70, 75, 76, 81], and by the fact that depressed women have a higher incidence of panic attacks [127] and co-morbid generalized anxiety disorder [128]. In contradiction of this theory is the finding that men, regardless of current depression status, exhibited higher right parietal activity than women with depression [125]. Interestingly, Stewart et al. found that women with MDD showed no differences in parietal activation compared to healthy controls [125]. However, women with a history of MDD demonstrated lower right parietal activation than women currently experiencing MDD, and this effect is greater following a large and recent intake of caffeine [125]. Similarly, a recent large intake of caffeine was found to emphasize the right parietal hyperactivation in men with depression, in comparison to healthy controls [125]. Therefore, it is possible that caffeine consumption may exacerbate symptoms of anxious arousal, such as panic, thereby highlighting the role of right parietal hyperactivation in MDD co-morbid with anxiety disorder [125].

Other network activity features have been examined under the context of sex differences in MDD, but to a much lesser extent. Increased theta activity within the ACC has been reported in patients with MDD, particularly within male patients [118], and a reduction in ACC theta activity appears to be indicative of a successful treatment response to sertraline, escitalopram, and venlafaxine in both male and female MDD patients [116]. A study looking at general global absolute power found that females with MDD had a significantly greater delta, theta, and beta power, compared to males with MDD, independent of depression severity scores [98]. Recently, Tement et al. demonstrated using EEG that male and female MDD patients have different alterations in brain connectivity [129]. Specifically, female patients with higher self-reported depression scores exhibited increased short-distance communication between the left posterior and central location [129]. As these networks overlap with those implicated in salience, these findings may suggest that females with depression have impaired perception of sensory data and therefore have biased information processing which increases the amount of perceived negative events [130]. Depressed males, on the other hand, with higher self-reported depression scores showed greater long-distance communication between left posterior regions and frontal/orbitofrontal locations [129], thought to be representative of a lack of cognitive control and enhanced rumination in male MDD patients [131]. Although not all findings are consistent, it is clear that sex influences aberrant neuronal circuitry in depression, which may hint at different pathophysiological pathways and/or differences in symptomatic expression of the disorder in males and females [132]. Regardless, it is evident that our understanding of this topic requires further investigation to clarify the discrepancy in findings, which are potentially attributable to the presence of co-morbid anxiety (as discussed above), circuit differences in patients with treatment resistant depression, and sex differences in skull thickness and general electrical properties of the brain, which may affect EEG measures [133–135]. Importantly, one must also consider the added complication of hormone fluctuations during female cycling and their potential influence on oscillatory activity in depressed women.

### MDD and the influence of sex hormones

Both clinical and preclinical studies have demonstrated a strong link between sex hormones and depression, although the exact relationship between the two is still unclear (for a full review, see [136]). In women, the prevalence of depression correlates with changes in hormonal fluctuations, such as puberty, prior to menstruation, during the postpartum period, and following the onset of menopause [2, 137]. Thus, it is likely that changes in ovarian hormones contribute to depression risk. In line with this, evidence supports the use of hormone replacement therapy (HRT; estradiol and progesterone combination) for the effective reduction of depression severity in perimenopausal and postmenopausal women [138, 139]. Moreover, 3 months of transdermal estrogen treatment reportedly increased alpha and theta power and decreased beta power in the temporal region in menopausal women with depression, although no effect on alpha asymmetry was found [140]. These changes reflected improved vigilance, a measure of an individual’s ability to adapt to the daily problems and life stressors, and subsequent improvement in scores on depression rating scales [140]. Furthermore, naturally cycling premenopausal women taking oral contraceptives (OC) were found to have reduced rates of depression and anxiety during the transition into menopause [141, 142], where a longer use of OC, and thus a longer duration of exposure to estrogens from menarche to menopause, was associated with a reduced risk of postmenopausal depression [142]. These results imply that ovarian hormones have a protective effect against affective disorders and that a decline in the levels of these sex hormones may contribute to one’s vulnerability to MDD development.

In men, a relationship between depression and a reduction in total or plasma concentrations of testosterone has been observed [143, 144]. Moreover, hypogonadal men with MDD who are insensitive to SSRI treatment showed improved antidepressant response following testosterone restitution [145, 146]. Interestingly, it is possible that the aromatase-catalyzed estrogen metabolite of testosterone confers the protective effects in males through estrogen receptors expressed throughout the male brain [147] and that men may have more consistent protection in their lifetime as testosterone and its metabolites do not fluctuate and cycle, as ovarian hormones do in females [2].

Animal models further support the relationship between sex hormones and a depression-like phenotype. OVX rats exhibited enhanced despair and anxiety-like behaviors, as measured by the forced swim test (FST) and elevated plus maze (EPM), respectively, and both were rescued with estradiol treatment [148–151]. An acute estradiol treatment was shown to produce antidepressant effects in intact male rats [152], as well as in both sexes of the clomipramine (CMI) rat model of depression [153]. It is likely that this effect was mediated by the interaction between estradiol and the HPA axis, as estradiol also has antianxiety and antidepressant effects in adrenalectomized female rats [151]. Interestingly, treatments of 5α-DHT and 3α,5α-androstanediol (3α-diol), metabolites of endogenous testosterone, in GNX male rats have also been found to reduce anxiety-like behavior [154, 155], as well as mediate the positive hedonic effect of testosterone [156, 157].

Remarkably, the direct influence of sex hormones and sex hormone manipulation on circuit function and its role in depression has not been examined extensively. However, based on the evidence to date, it is clear that this area of research merits further investigation and may help elucidate the mechanism involved in sex-specific susceptibility to depression.

### Female cycling and its effect on circuit function in MDD

As previously mentioned, women most commonly exhibit symptoms of depression and are at a higher risk of depression during periods associated with changes in gonadal hormone levels [2, 137]. Moreover, ovarian hormone fluctuations during the menstrual cycle have been found to influence depressive symptoms. Women with premenstrual depressive disorder (PMDD) express exacerbated symptoms of dysthymia, irritability, anxiety, and impaired working and emotional memory specifically during the luteal phase, when progesterone levels are highest [158, 159]. Additionally, PMDD patients show luteal-dependent negative bias in information processing [160, 161], increase in negative affect, and impairments in memory task performance [159]. Therefore, it appears that the fluctuations in ovarian hormones may be associated with the female vulnerability to depression. Importantly, menopausal women with and without depression have no difference in plasma estrogen concentration [162], suggesting that estrogen levels are not the sole contributor to depression expression and symptomology [163]. This is further supported when considering that both hormone cycling and the loss of hormones during menopause are normal physiological processes; however, some women have a negative affective response to these hormonal changes. Thus, it is likely that there are other underlying pathological mechanisms contributing to a woman’s inability to properly respond and adapt to these physiological hormone fluctuations. Overall, it is apparent that the mechanisms underlying the connection between ovarian hormone cycling and affective disorders are complex, and based on previous evidence, likely involve cycling-mediated changes in neuronal circuitry. However, very few studies have investigated the influence of cycling on network activity in depressed females.

Zhang et al. reported that the degree of frontal asymmetry positively correlated to self-reported depression severity score only during the periovulation phase in naturally cycling women [164]. Furthermore, the level of frontal asymmetry during the premenstrual phase was predictive of a woman’s postmenstrual depression severity score, while the degree of frontal asymmetry during the postmenstrual and periovulation phases predicted depression severity scores at periovulation [164]. Thus, as alpha asymmetry is hypothesized to be associated with symptoms of anhedonia and reduced motivation [62], the menstrual cycle may modulate depressive symptoms through its interaction with frontal alpha activity [164]. Frontal asymmetry has also been reported in menopausal women with depression and was positively correlated with depression severity scores [162]. Additionally, the authors found that menopausal women with depression exhibited reduced global total power and absolute power in the delta, theta, and beta bands, as well as increased global relative delta and theta power, accompanied by attenuated alpha power [162]. Collectively, these changes in network circuitry are associated with reduced vigilance in these patients [162]. Moreover, although estradiol levels did not directly correlate with depression severity in menopausal women, they did positively correlate with the EEG changes linked to decreased vigilance, and these EEG changes directly correlated with depression severity scores [162]. Therefore, when the findings are taken together, it is likely that the menopause-associated decline in estradiol contributes to circuitry changes, which subsequently manifest symptomatically (i.e., reduced vigilance), and in turn, contribute to the development of depression [162].

These studies have only begun to scratch the surface of our understanding of the menstrual cycle’s influence on circuitry changes and its implications for mood and depression, and they highlight the importance of continued investigation in this field. Thus, the literature points to a need for a greater focus on the neurobiological influence of female cycling, as well as an increased incorporation of female animals in preclinical experimental designs. Current evidence of the role of female sex hormones in MDD and circuit function is summarized in Table 3.

**Table 3:** The role of female sex hormones in MDD overall and in MDD circuit function

Study	Subjects/treatment	Major findings
Rudolph et al. [138]	Postmenopausal women with and without MDD Treatment: 24 weeks of continuous combined HRT (2 mg estradiol valerate + 2 mg dienogest)	A clinically relevant reduction in depression severity
Marsh et al. [142]	Regularly cycling premenopausal women Treatment: various durations of OC	A longer use of OC (i.e., a longer exposure to estrogens from menarche to menopause) was associated with reduced risk of postmenopausal depression.
Saletu et al. [140]	Menopausal women with and without MDD Treatment: 3 months of transdermal estradiol (100 μg/week)	Estradiol treatment increased alpha and theta power and reduced beta power in the temporal region, which reflected improved vigilance. No changes in frontal alpha asymmetry observed.
Zhang et al. [164]	Regularly cycling women with and without MDD	The degree of frontal asymmetry positively correlated to depression severity scores during periovulation only. The level of frontal asymmetry during the premenstrual phase was predictive of postmenstrual depression severity scores. The degree of frontal asymmetry during postmenstrual and periovulation phases predicted depression severity scores during periovulation.
Saletu et al. [162]	Postmenopausal women without previous HRT, with or without MDD	Frontal asymmetry positively correlates with depression severity scores. Postmenopausal women with depression had reduced global total and absolute power in delta, theta, and beta bands, as well as increased global relative delta and theta power, and attenuated alpha power. Both estradiol levels and depression severity scores directly correlated with EEG changes.

*HRT* hormone replacement therapy, *OC* oral contraceptives

## Conclusions

About twice as many women suffer from MDD than men, and it is well established that females are more vulnerable to the effects of stress, a neurobiological response that mediates neuronal adaptations tightly linked to depression [165]. However, the underlying pathological mechanisms of depression and sex-specific differences in these mechanisms that contribute to the female susceptibility to MDD are poorly understood largely due to the high degree of inconsistencies in the literature and the number of potential confounding variables (i.e., co-morbid anxiety, hormone status, treatment responsiveness). Although there is an evident link between estrogens and mood, it is critical to emphasize that not all cycling females develop MDD, while many males do develop chronic depression, despite their lack of hormone fluctuations. As such, there are clearly other contributors to MDD vulnerability in addition to the influence of sex steroids. This further highlights the pressing need for additional research to address these disparities and gaps in the literature regarding this topic. Importantly, additional EEG and imaging studies are required to delineate the sex differences in MDD-associated oscillations and network activity, while at the same time accounting for co-morbid anxiety and antidepressant treatment resistance. These studies will help elucidate the influence of female cycling and menopause on network activity in the context of MDD and further our understanding of the specific roles of estradiol and progesterone. In addition, other contributors to the pathology should be considered, such as genetic and physiological factors that influence how males and females differentially respond to environmental stressors. A better understanding of how sex hormones influence neuronal circuit function in the context of depression is a necessary starting point in the search for the neuropathological mechanisms involved in depression susceptibility.

## Abbreviations

3α-diol: 3α,5α-Androstanediol
5α-DHT: 5α-Dihydrotestosterone
ACC: Anterior cingulate cortex
CMI: Clomipramine
DBS: Deep brain stimulation
EEG: Electroencephalography
EPM: Elevated plus maze
fMRI: Functional magnetic resonance imaging
FST: Forced swim test
GNX: Gonadectomized
HIP: Hippocampus
HPA: Hypothalamic-pituitary-adrenal
HRT: Hormone replacement therapy
LTP: Long-term potentiation
MDD: Major depressive disorder
MEG: Magnetoencephalography
NAc: Nucleus accumbens
OC: Oral contraceptives
OCD: Obsessive-compulsive disorder
OVX: Ovariectomized
PFC: Prefrontal cortex
PMDD: Premenstrual depressive disorder
PV: Parvalbumin
SSRI: Selective serotonin reuptake inhibitor
SST: Somatostatin
TP: Testosterone propionate
VTA: Ventral tegmental area
